# Supplementary material for: Microglia clear neuron-released α-synuclein via selective autophagy and prevent neurodegeneration
Source: Nat Commun. 2020 Mar 13;11:1386. doi: 10.1038/s41467-020-15119-w (PMC7069981; doi:10.1038/s41467-020-15119-w)
Supplement: Supplementary file 1 — Supplementary Information [file 41467_2020_15119_MOESM1_ESM.pdf]

Supplementary Information

Title: Microglia Clear Neuron-released α-Synuclein via Selective Autophagy and Prevent Neurodegeneration

Choi et al.

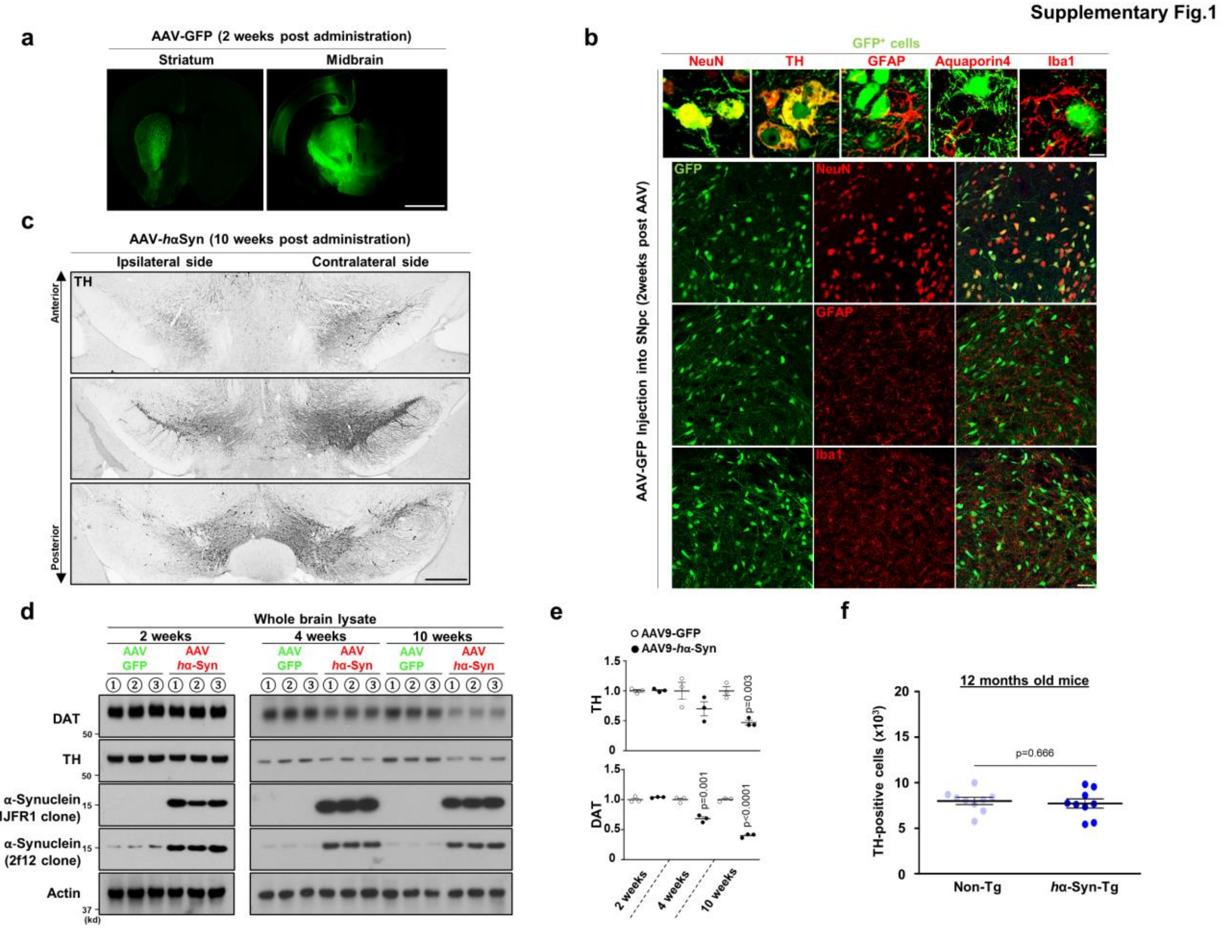

Supplementary Figure 1. Characterization of two different PD model overexpressing human α-synuclein.

(a) AAV9 expressing GFP under the neuron-specific synapsin promoter (AAV-GFP) was administered into substantia nigra and GFP expression was examined after 2-weeks. Scale bar, 2mm.

(b) To confirm the neuron-specific expression of GFP, brain slices were stained with antibodies against neuronal proteins (NeuN and Tyrosine hydroxylase (TH)), astrocytes markers (GFAP and Aquaporin 4), and microglia marker (Iba-1). Scale bar, 5 µm in the upper panel, 50 µm in the lower panel.

(c) Loss of dopaminergic neurons in the ipsilateral side of SNpc was visualized by tyrosine hydroxylase (TH) staining with DAB staining method in three different coronal sections at 10-weeks post-AAV-*h*αSyn administration. Scale bar, 500 µm.

(d, e) After AAV-GFP (n=3, each group) or AAV-*h*α-Syn (n=3, each group) administration, whole brains were homogenized and assayed for W.B using antibodies against DAT (p=0.001 and 2.07E-05 at 4 and 10 weeks, respectively), TH (p=0.03 at 10weeks), and α-synuclein at indicated time (d). Actin was used as loading controls. The intensities of protein bands were quantified (e). *p* values were calculated by unpaired two-tailed Student's t-test.

(f) Brains of 1-year-old non-Tg (n=9) and *h*α-Syn-Tg mice (n=9) aged mice per genotype were fixed and stained using an antibody against tyrosine hydroxylase (TH) using DAB method. TH^{+}-positive cells were counted. *p* values were calculated by unpaired two-tailed Student's t-test.

All values are reported as mean ± SEM. Data are representative of more than three different animals.

Supplementary Fig.2

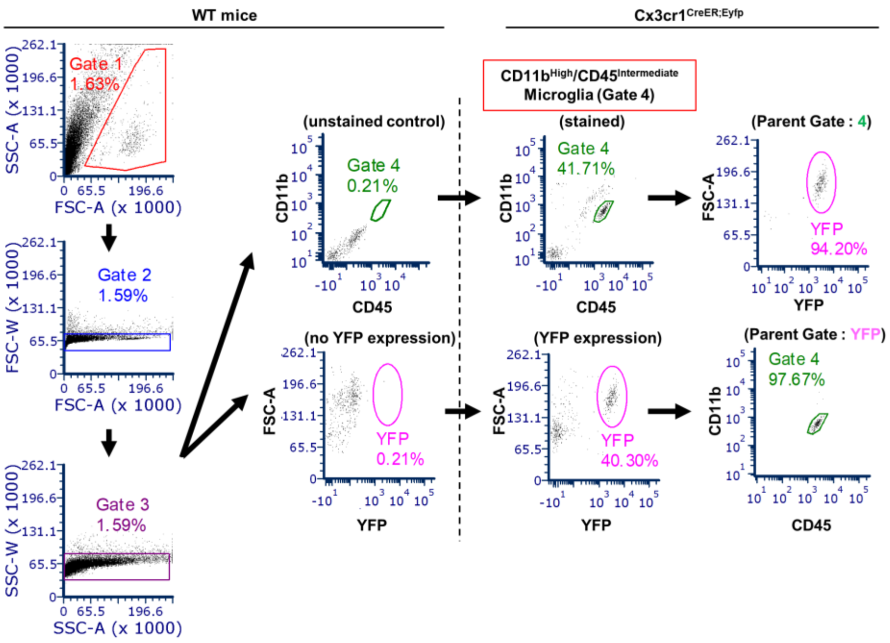

Supplementary Figure 2. Gating strategy for obtaining CD45^{intermediate} and CD11b^{high} microglia from adult mouse brain.

Enriched microglia population prepared from a Percoll®-gradient method were stained with CD45 and CD11b antibodies conjugated with APC-Cy7 and Percp-Cy5.5, respectively. Microglia cell population were gated at the first (Gate 1) and gated for single cell population (Gate 2 and 3). CD45^{intermediate} and CD11b^{high} cells (Gate 4 population) were collected. See the detail information in Method.

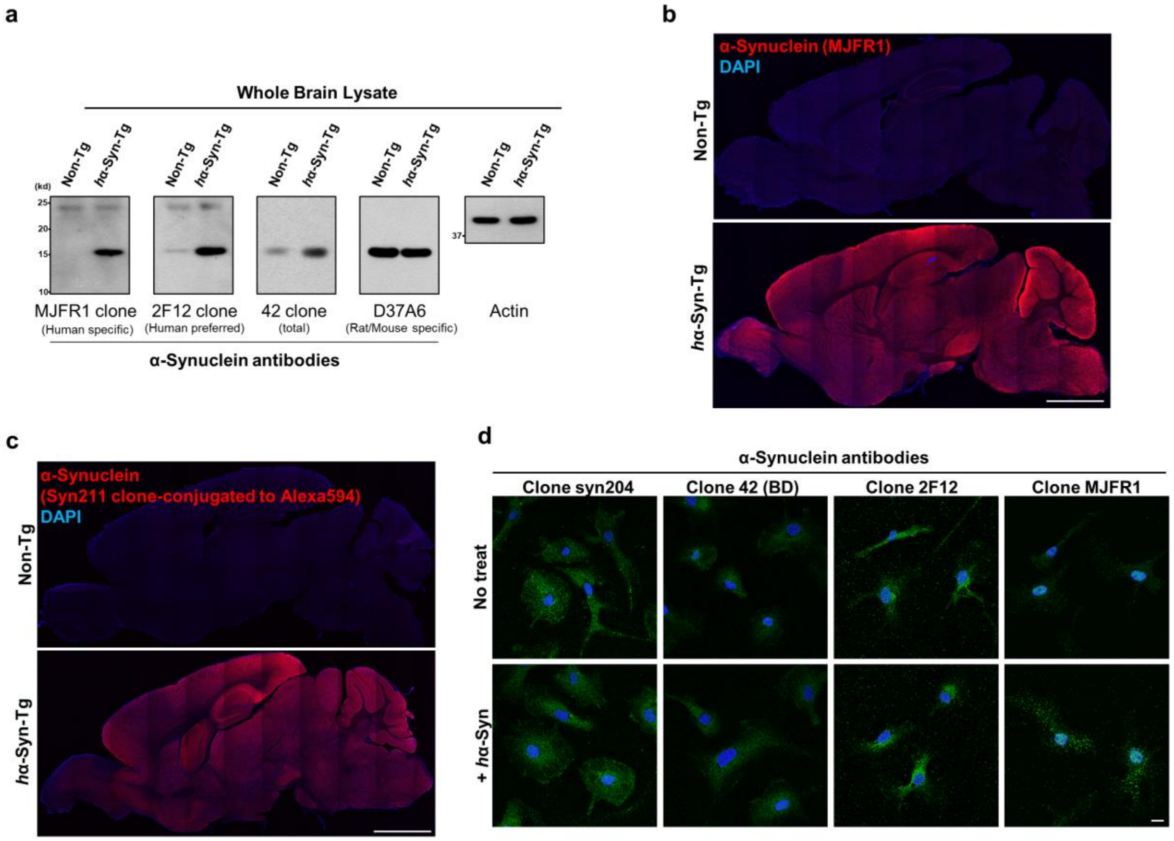

Supplementary Figure 3. Test the specificity of various α-synuclein antibodies.

(a-c) Brains from 3-months old non-Tg and *h*α-Syn-Tg mice were homogenized in TBS/Sucrose buffer and assayed for W.B with indicated antibodies against α-synuclein (a) or fixed and stained with human α-synuclein antibodies (MJFR1 clone in b; Syn211 clone in c). Actin was used as a loading control. Scale bar, 2mm.

(d) Cultured primary microglia were stained with indicated human α-synuclein antibodies in the presence/absence of 250nM recombinant human α-synuclein treatment for 1 hour followed by Alexa488-secondary antibody. Scale bar, 10μm.

Data are representative of three independent experiments.

Supplementary Fig.4

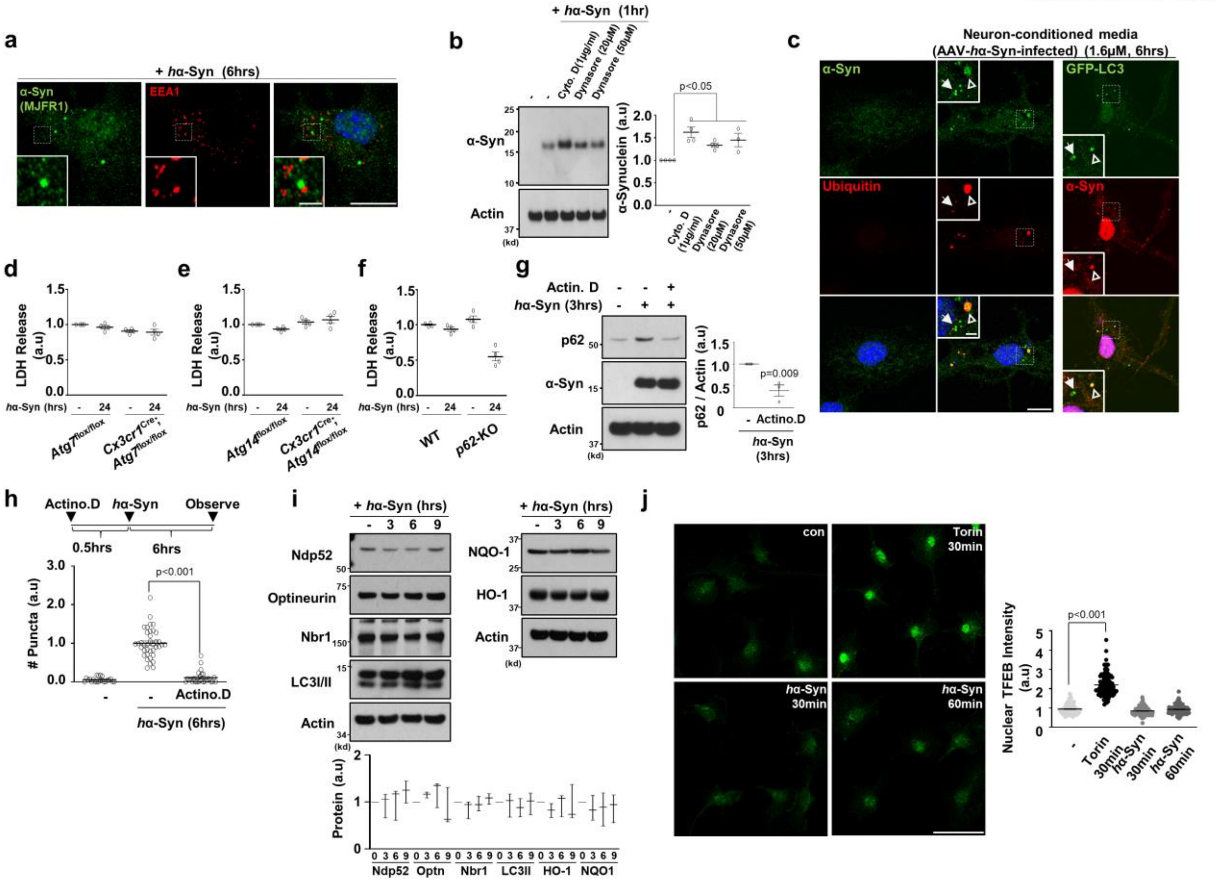

Supplementary Figure 4. Characterization and effects of human α-synuclein on cultured microglia.

(a) Microglia were treated 250nM *h*α-Syn protein for 6 hours and stained using antibodies against human α-synuclein (MJFR1 clone) and EEA1. Scale bar, 10 μm; 5 μm in magnified boxes.

(b) Microglia were pretreated with cytochalasin D (Cyto. D), an inhibitor of actin polymerization, and Dynasore, an inhibitor of endocytosis, before adding 250nM *h*α-Syn for 1 hour. Band intensities were quantified in the right panel. Acin was used a loading control. *P* values were calculated by One-way ANOVA with Newman–Keuls post hoc test. $F(3,11)=9.499$, $p=0.0022$.

(c) Embryonic mice cortical neuron was prepared and infected AAV9-α-*h*Syn (10µl of $6x10^{13}$ GC/ml virus into 2ml medium) at DIV3 and the medium (containing ~3.2µM of *h*Syn) was collected at DIV14. Cultured wt microglia (left panel) and GFP-LC3 microglia (right panel) were treated with neuron-conditioned medium by exchanging the half of medium for 6 hours, and stained. Scale bar, 10µm; 2µm in the magnified box.

(d-f) The levels of lactate dehydrogenase (LDH) were measured using LDH-Cytotoxicity Colorimetric Assay Kit (#K311, Biovision, Mountain View, CA) according the manufacturer's instructions in the medium collected from cultured microglia either from $Atg7^{\text{flox/flox}}$ mice (**d**) or $Atg14^{\text{flox/flox}}$ mice (**e**) with or without $Cx3cr1^{\text{Cre}}$, and *p62*-KO mice (**f**) in the absence/presence of 250nM *h*α-Syn protein. n=3 over three independent experiments.

(g, h) Microglia were pretreated with Actinomycin D, a blocker of mRNA translation, for 30 minutes and treated with 250nM *h*α-Syn protein. Levels of p62 protein were quantified in the lower panel. The number of *h*α-Syn/ubiquitin-positive puncta was counted and quantified (g). *P* values were calculated by Unpaired two-tailed Student's t-test (**g,** p=0.009) and One-way ANOVA with Newman–Keuls post hoc test (**h**, F(2, 84)=126.3, p= 4.79E-26). n=3 (g) and n=22 for con, n=38 for *h*α-Syn, and n=27 for *h*α-Syn with Actino.D (h) over three independent experiments.

(i) After 250nM *h*α-Syn protein treatment, cells were lysed and assayed for W.B using antibodies against NDP52, Optineurin, NBR1, LC3 I/II, NQO-1, and HO-1. Actin was used as a loading control. Band intensities were quantified in the lower panel. n=3 over three independent experiments.

(j) After 250nM *h*α-Syn protein treatment or Torin, cells were fixed and stained using antibody against TFEB. Intensities of Nuclear TFEB were measured. n=97 for con, n=95 for Torin, n=114

for *h*α-Syn 30min, and n=126 for *h*α-Syn 60min over three independent experiments. *P* values were calculated by One-way ANOVA with Newman–Keuls post hoc test. $F(3,428)=355.7$, $p= 7.85E-116$. Scale bar, 50µm.

All values are reported as mean ± SEM. Data are representative of at least three independent experiments.

Supplementary Fig.5

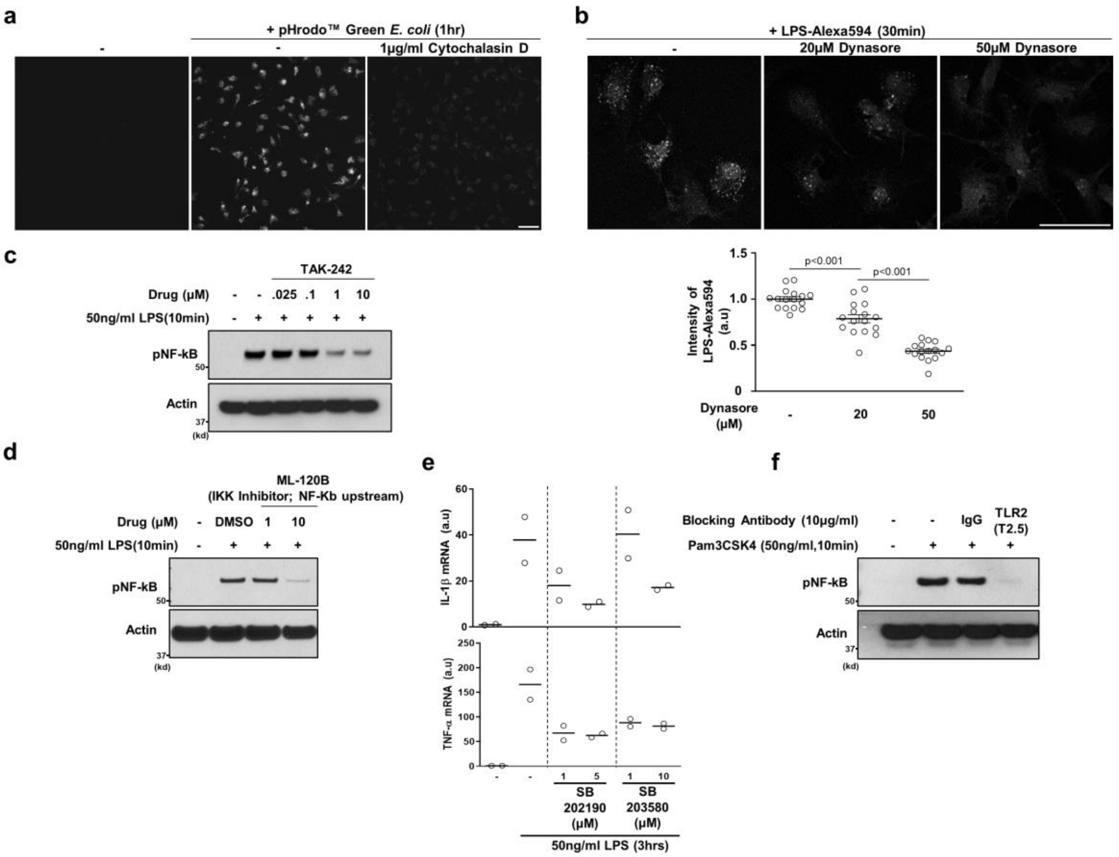

Supplementary Figure 5. Drugs and TLR2 blocking antibody test.

(a, b) Microglia were treated with Cytochalasin D (a) and Dynasore (b) as indicated concentrations before adding pHrodo™ Green *E.coli* (#P35366, Thermofisher Scientific) (a) and LPS conjugated with Alexa594 (L-23353, Thermofisher Scientific) (b), respectively, according the manufacturer's instructions. p values were calculated by One-way ANOVA with Newman–Keuls post hoc test (b, $F(2,45)=75.67$, $p= 4.02E-15$). Scale bar, 50 μm. Images are representative of three independent experiments.

(c-e) Microglia were pretreated with TAK242 (a), ML-120B (b, an IKK2 inhibitor, #4899, Tocris), SB203580 and SB202190 (c, two different p38 inhibitors, #1202 and #1264, Tocris) before adding

50ng/ml LPS (L7770, Sigma), a TLR4-specific agonist, for indicated time. The protein levels of p-NF-κB (**c, d**) and mRNA levels of *Il-1b* and *Tnf* (**e**) were examined by W.B and RT-qPCR, respectively. n=2 (e) over two independent experiments for drug validation.

(f) Cells were pretreated with 10 μg/ml of TLR2 blocking antibody (T2.5, #121802, Biolegend, San Diego, CA) for 10 minutes and treated with Pam_3CSK_4 (Invivogen, San Diego, CA), a TLR2-specific agonist, for 10 minutes.

All values are reported as mean ± SEM.

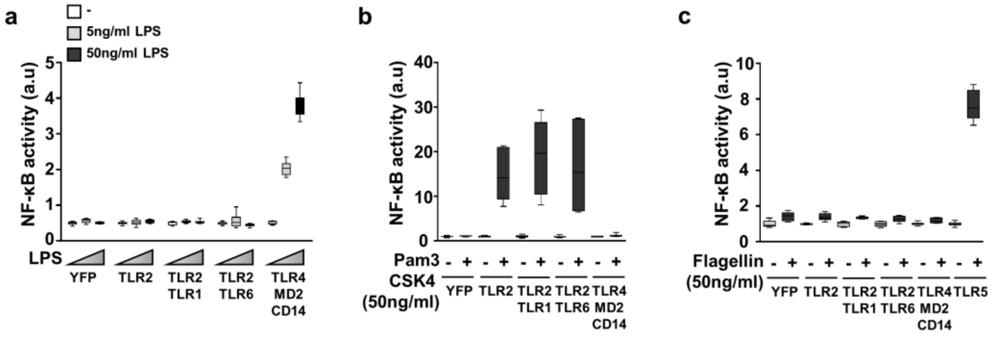

Supplementary Figure 6. Test of reconstituted TLR system in HEK293 cells by specific agonists.

HEK293T cells were transfected with a luciferase vector that expresses luciferase under the promoter containing NF-κB-binding elements, and various TLRs for 1 day. Then, cells were treated with LPS with indicated concentration (**a**), 50ng/ml Pam_3CSK_4 (**b**), or 50ng/ml recombinant Flagellin (**c**). After 24 hours later, the activity of luciferase was measured by ONE-Glo™ Luciferase Assay System according to the manufacturer's instructions. See the detail in Method. All values are reported as mean ± SEM. n=6 over two independent experiments for validation.

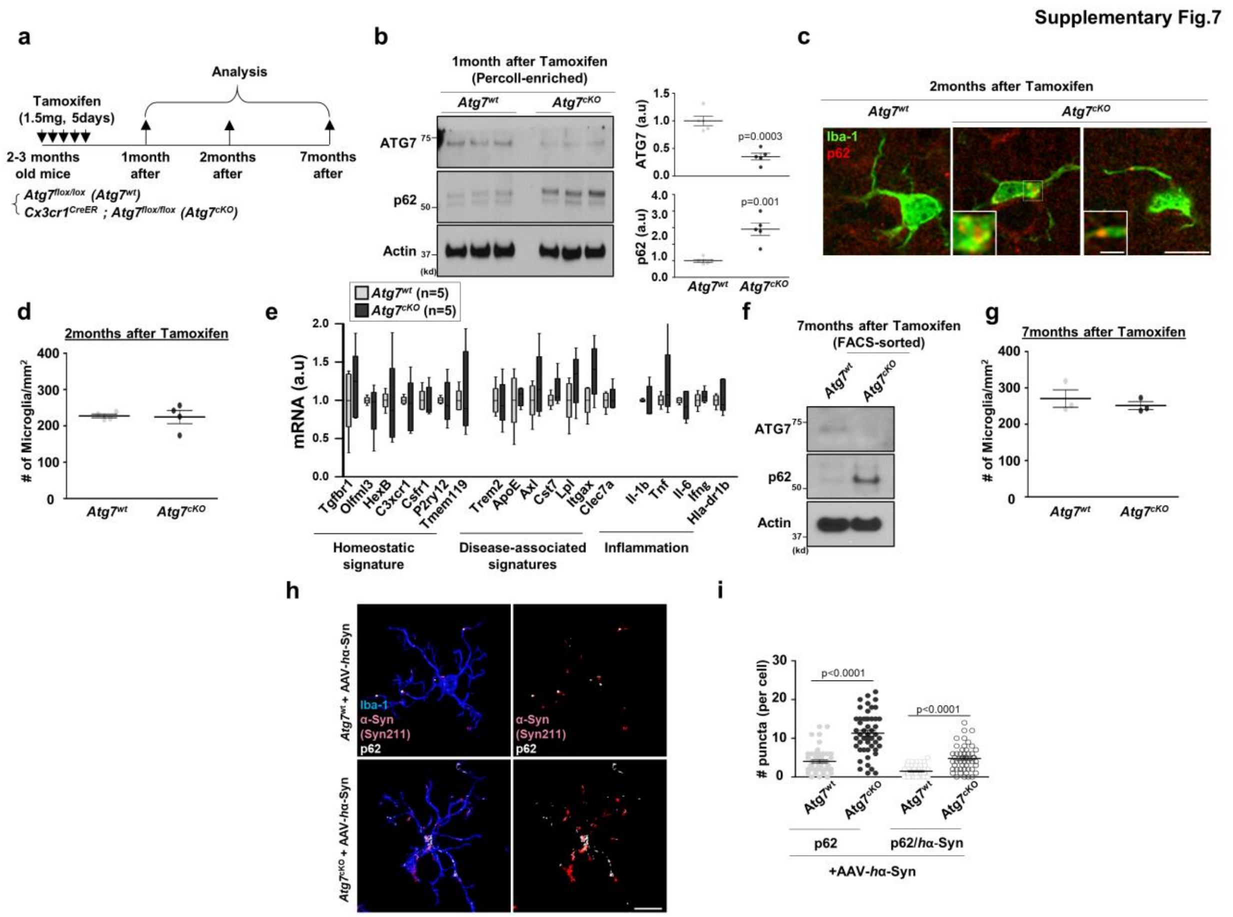

Supplementary Figure 7. Establishment of microglia-specific *Atg7*-deficient mice.

(a) Experimental plan for obtaining microglia-specific *Atg7*-deficient mice by injecting tamoxifen into *Atg7*$^{\text{flox/flox}}$ mice and *Cx3cr1*$^{\text{CreER-IRES-Eyfp}}$; *Atg7*$^{\text{flox/flox}}$ mice

(b) At 1-month after tamoxifen, the microglia-enriched population was collected using Percoll-gradient method and lysed for W.B using antibodies against ATG7 and p62. Actin was used as a loading control. Intensities of protein bands were quantified in the right panel. n=5 per group. *p* values were calculated by Unpaired two-tailed Student's t-test.

(c, d) At 2-months after tamoxifen, brain sections (n=4 per group) were fixed and stained with antibodies using Iba-1 and p62 (**c**). The number of Iba-1-positive microglia at the cortex was

counted using ImageJ software (NIH, Bethesda, MD) (**d**). Scale bar, 10μm; 2μm in magnified images. Images are representative of three independent experiments.

(e) At 2-months after tamoxifen, $CD45^{intermediate}$ and $CD11b^{high}$ microglia were isolated from brains of *Atg7*$^{flox/flox}$ mice (n=5) and *Cx3cr1*$^{CreER\text{-}IRES\text{-}Eyfp}$; *Atg7*$^{flox/flox}$ mice (n=5), and assayed for RT-qPCR using primers for genes associated with homeostatic signatures, disease-associated signatures, and inflammation.

(f) At 7-months after tamoxifen, pooled $CD45^{intermediate}$ and $CD11b^{high}$ microglia were isolated by flow cytometry from brains of *Atg7*$^{flox/flox}$ mice (n=2) and *Cx3cr1*$^{CreER\text{-}IRES\text{-}Eyfp}$; *Atg7*$^{flox/flox}$ mice (n=2), and assayed for W.B using antibodies against ATG7 and p62. Actin was used as a loading control.

(g) At 7-months after tamoxifen, Iba-1-positive microglia in the cortex were counted in Atg7$^{flox/flox}$ mice (n=3) and *Cx3cr1*$^{CreER\text{-}IRES\text{-}Eyfp}$; *Atg7*$^{flox/flox}$ mice (n=3).

(h, i) After 6 weeks after AAV, brain slices were fixed and stained with antibodies against human α-synuclein, p62, and Iba-1. Representative 3D reconstruction pictures of microglia containing p62 and human α-synuclein in the striatum were produced using Imaris (Bitplane, **h**). The number of p62 puncta or p62-positive/*h*α-Syn-positive puncta was quantified in microglia at striatum of *Atg7*$^{flox/flox}$ mice (n=3) and *Cx3cr1*$^{CreER\text{-}IRES\text{-}Eyfp}$; *Atg7*$^{flox/flox}$ mice (n=3) (**i**). *p* values ($p<0.0001$) were calculated by two-tailed Mann–Whitney U test. Scale bar, 10μm. Images are representative of three independent experiments. All values are reported as mean ± SEM.

Supplementary Fig. 8

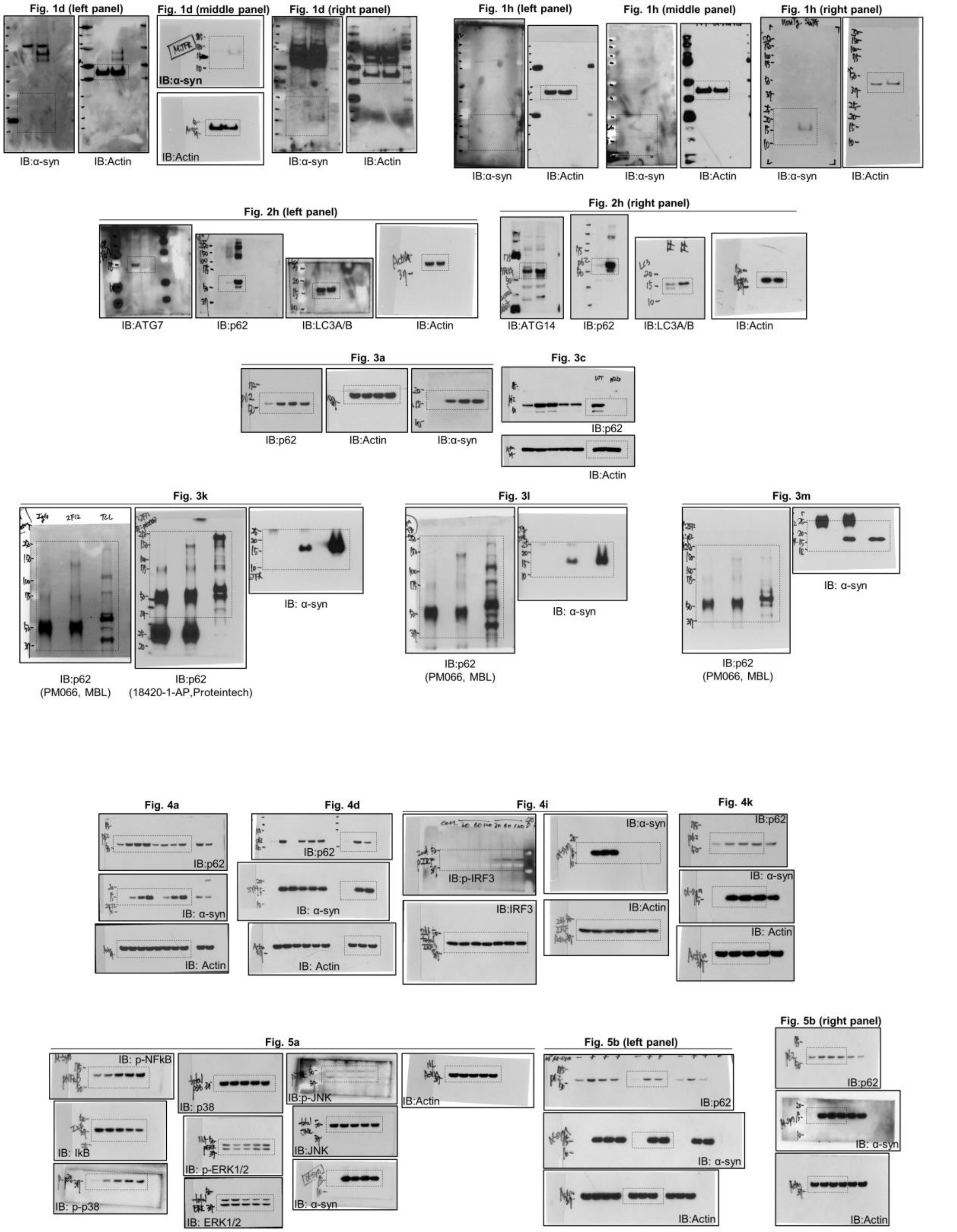

Fig. 6b

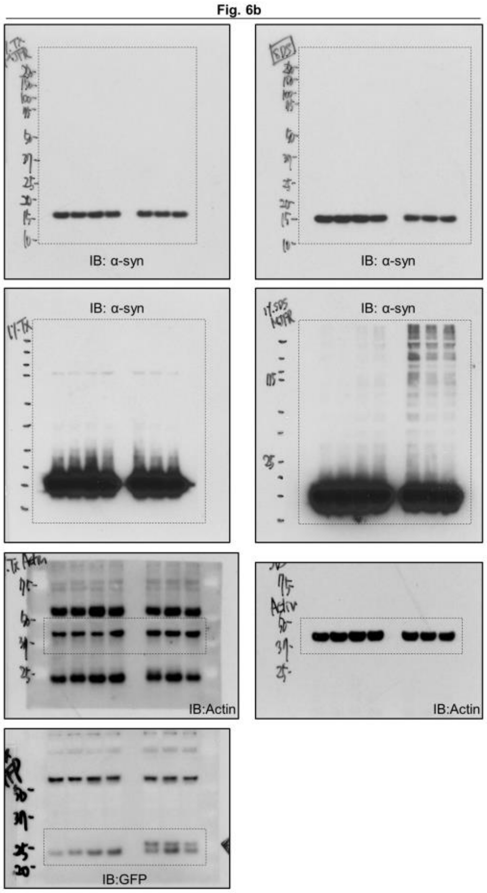

Fig. 6f

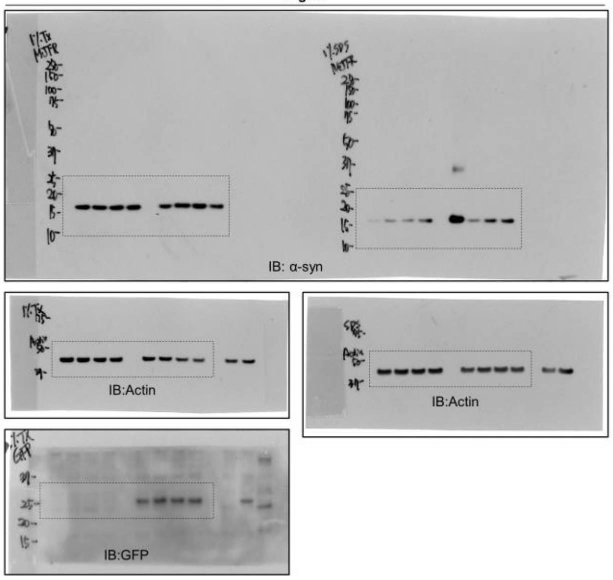

Supplementary Figure 8. Uncropped blot images
