# Supplementary material for: Microglia clear neuron-released α-synuclein via selective autophagy and prevent neurodegeneration
Source: Nat Commun. 2020 Mar 13;11:1386. doi: 10.1038/s41467-020-15119-w (PMC7069981; doi:10.1038/s41467-020-15119-w)
Supplement: Supplementary file 2 — Reporting Summary [file 41467_2020_15119_MOESM2_ESM.pdf]

nature research

Corresponding author(s): Zhenyu Yue

Last updated by author(s): Feb 6, 2020

# Reporting Summary

Nature Research wishes to improve the reproducibility of the work that we publish. This form provides structure for consistency and transparency in reporting. For further information on Nature Research policies, see Authors & Referees and the Editorial Policy Checklist.

## Statistics

For all statistical analyses, confirm that the following items are present in the figure legend, table legend, main text, or Methods section.

| n/a | Confirmed | |
|---|---|---|
| ☐ | ☒ | The exact sample size ($n$) for each experimental group/condition, given as a discrete number and unit of measurement |
| ☐ | ☒ | A statement on whether measurements were taken from distinct samples or whether the same sample was measured repeatedly |
| ☐ | ☒ | The statistical test(s) used AND whether they are one- or two-sided<br>*Only common tests should be described solely by name; describe more complex techniques in the Methods section.* |
| ☐ | ☒ | A description of all covariates tested |
| ☐ | ☒ | A description of any assumptions or corrections, such as tests of normality and adjustment for multiple comparisons |
| ☐ | ☒ | A full description of the statistical parameters including central tendency (e.g. means) or other basic estimates (e.g. regression coefficient) AND variation (e.g. standard deviation) or associated estimates of uncertainty (e.g. confidence intervals) |
| ☐ | ☒ | For null hypothesis testing, the test statistic (e.g. $F$, $t$, $r$) with confidence intervals, effect sizes, degrees of freedom and $P$ value noted<br>*Give P values as exact values whenever suitable.* |
| ☒ | ☐ | For Bayesian analysis, information on the choice of priors and Markov chain Monte Carlo settings |
| ☒ | ☐ | For hierarchical and complex designs, identification of the appropriate level for tests and full reporting of outcomes |
| ☒ | ☐ | Estimates of effect sizes (e.g. Cohen's $d$, Pearson's $r$), indicating how they were calculated |

*Our web collection on statistics for biologists contains articles on many of the points above.*

## Software and code

Policy information about availability of computer code

| | |
|---|---|
| Data collection | Imaging : Zen 2011 software (v2.6), Zeiss Axioplan2 (v4.9.1)<br>Flow cytometry: FACSDiva (v8.0.1) |
| Data analysis | Fiji (ImageJ for image analysis), Stereo Investigator (v 2017, for TH/Nissl counting), Imaris (for representative image generation in vivo), GraphPad Prism v8.3.1 (for quantification and statistical analysis) |

For manuscripts utilizing custom algorithms or software that are central to the research but not yet described in published literature, software must be made available to editors/reviewers. We strongly encourage code deposition in a community repository (e.g. GitHub). See the Nature Research guidelines for submitting code & software for further information.

## Data

Policy information about availability of data

All manuscripts must include a data availability statement. This statement should provide the following information, where applicable:

- Accession codes, unique identifiers, or web links for publicly available datasets
- A list of figures that have associated raw data
- A description of any restrictions on data availability

The authors declare that all data supporting the findings of this study are available within the paper and its supplementary information files.

# Field-specific reporting

Please select the one below that is the best fit for your research. If you are not sure, read the appropriate sections before making your selection.

☒ Life sciences ☐ Behavioural & social sciences ☐ Ecological, evolutionary & environmental sciences



October 2018

For a reference copy of the document with all sections, see nature.com/documents/nr-reporting-summary-flat.pdf

# Life sciences study design

All studies must disclose on these points even when the disclosure is negative.

**Sample size**
No statistical method was used in deciding sample sizes. The sample size per group was determined from previous publications with similar methodologies (Kam et al., 2018, Science, for animal number of PD model; Itakura et al., 2011, JCB, for autophagosome-related puncta counting).

**Data exclusions**
We didn't exclude any sample.

**Replication**
Replication of experiments was successful in three different experiments/cohort and data is accumulated during the analysis process. Results also were replicated in independent experimental setting by different authors.

**Randomization**
All cells analyzed for the quantification were randomly selected. Control mice and their littermate mutant/KO mice were collected from each cohort depending on the genotype and used for the experiment. WT mice injected with either AAV-GFP or AAV-Syn were randomly assigned into different time points.

**Blinding**
All TH/Nissl counting experiments were confirmed by a blinded test. Also, cell assay experiments including NFkB-luciferase and imaging were performed by more than two people in a blinded test.  Protein analyses using Western blotting were not performed blind to load samples by order.

# Reporting for specific materials, systems and methods

We require information from authors about some types of materials, experimental systems and methods used in many studies. Here, indicate whether each material, system or method listed is relevant to your study. If you are not sure if a list item applies to your research, read the appropriate section before selecting a response.

## Materials & experimental systems

| n/a | Involved in the study |
|---|---|
| ☐ | ☒ Antibodies |
| ☐ | ☒ Eukaryotic cell lines |
| ☒ | ☐ Palaeontology |
| ☐ | ☒ Animals and other organisms |
| ☒ | ☐ Human research participants |
| ☒ | ☐ Clinical data |

## Methods

| n/a | Involved in the study |
|---|---|
| ☒ | ☐ ChIP-seq |
| ☐ | ☒ Flow cytometry |
| ☒ | ☐ MRI-based neuroimaging |

## Antibodies

**Antibodies used**
Aquaporin 4 (AB3594, Millipore), GFAP (#130300, Thermofisher Scientific), GFP/YFP (MA5-15256, Thermofisher Scientific), Iba-1 (019-19741, Wako, Japan), NeuN (MAB377, Chemicon), p62 (GP62-C, Progen, Germany), α-synuclein (clone MJFR1, ab138501, Abcam; clone syn211 conjugated with Alexa594 fluorescein, sc-12767 AF594, Santa Cruz Biotechnology), ubiquitin (clone P4D1, sc-8017, Santa Cruz Biotechnology), Tyrosine hydroxylase (AB152, Millipore), pS129 α-synuclein (ab51253, Abcam), EEA1 (610457, BD Bioscience), Actin (#3700S, Cell Signaling), ATG7 (MAB6608, R&D), ATG14 (PD026, MBL), Dopamine transporter (MAB369, Millipore), ERK1/2 (#9107S, Cell Signaling), p-ERK1/2 (Thr202/Tyr204, #4370P, Cell Signaling), IκB (#4814, Cell Signaling), p-IRF3 (S396, #29047, Cell Signaling), IRF3 (#4302, Cell Signaling), JNK (#9252S, Cell Signaling), p-JNK (Thr183/Tyr185, #4671S, Cell Signaling), LC3B (#2775S, Cell Signaling), NBR1 (16004-1-AP, Proteintech), NDP52 (12229-1-AP, Proteintech), p-NF-κB (S536, #3033S, Cell Signaling), Optineurin (10837-1-AP, Proteintech), p38 (#9212S, Cell Signaling), p-p38 (Thr180/Tyr182, #4511S, Cell Signaling), p62 (PM066, MBL), Tyrosine hydroxylase (T2928, Sigma), mouse CD16/CD32 (#14-0161-82, Invitrogen), CD45 Monoclonal Antibody (I3/2.3) conjugated with APC-Cyanine7 (#A15395, Invitrogen), CD11b Monoclonal Antibody (M1/70) conjugated with PerCP-Cyanine5.5 (#45-0112-82, Invitrogen), TFEB (A303-673A, Bethyl Laboratories Inc, TX), NQO-1 (11451-1-AP, Proteintech), HO-1 (sc-10789, Santa Cruz), Goat anti-Rabbit IgG (H+L) Highly Cross-Adsorbed Secondary Antibody, Alexa Fluor 488 (A11034, Invitrogen), Goat anti-Mouse IgG (H+L) Highly Cross-Adsorbed Secondary Antibody, Alexa Fluor 594 (A-11032, Invitrogen), Goat anti-Guinea Pig IgG (H+L) Highly Cross-Adsorbed Secondary Antibody, Alexa Fluor 647 (A-21450, Invitrogen)

**Validation**
Aquaporin 4 (AB3594, Millipore)-PMID:30561329
GFAP (#130300, Thermofisher Scientific, on mouse tissue) - https://www.thermofisher.com/antibody/product/GFAP-Antibody-clone-2-2B10-Monoclonal/13-0300
GFP/YFP (MA5-15256, Thermofisher Scientific) - https://www.thermofisher.com/antibody/product/GFP-Antibody-clone-GF28R-Monoclonal/MA5-15256
Iba-1 (019-19741, Wako, Japan, on mouse brain) - https://labchem-wako.fujifilm.com/us/category/01213.html
NeuN (MAB377, Chemicon, on mouse tissue) - https://www.emdmillipore.com/US/en/product/Anti-NeuN-Antibody-clone-A60,MM_NF-MAB377
p62 (GP62-C, Progen, Germany, on mouse tissue) - https://www.progen.com/anti-p62-sqstm1-c-terminus-guinea-pig-polyclonal-serum.html
α-synuclein (clone MJFR1, ab138501, Abcam) - https://www.abcam.com/alpha-synuclein-antibody-mjfr1-ab138501.html, further

validated in supplementary figure3
α-synuclein (clone syn211 conjugated with Alexa594 fluorescein, sc-12767 AF594, Santa Cruz Biotechnology) - validated in supplementary figure3
ubiquitin (clone P4D1, sc-8017, Santa Cruz Biotechnology, on mouse brain) - PMID: 29362455
Tyrosine hydroxylase (AB152, Millipore, on mouse cell) - https://www.sigmaaldrich.com/catalog/product/mm/ab152?lang=en&region=US
pS129 α-synuclein (ab51253, Abcam, comparison among many pS129 antibodies) - PMID:29888794
EEA1 (610457, BD Bioscience, on mouse cell) - PMID:29695488
ATG7 (MAB6608, R&D) - validated in Figure 2h in cKO mouse cells
ATG14 (PD026, MBL) - validated in figure 2h in cKO mouse cells
Dopamine transporter (MAB369, Millipore) - PMID:23481388
TFEB (A303-673A, Bethyl Laboratories Inc, TX) - PMID:27171064
HO-1 (sc-10789, Santa Cruz) - PMID:27869147
Actin (#3700S, Cell Signaling) - https://www.cellsignal.com/products/primary-antibodies/b-actin-8h10d10-mouse-mab/3700
ERK1/2 (#9107S, Cell Signaling) - https://www.cellsignal.com/products/primary-antibodies/p44-42-mapk-erk1-2-3a7-mouse-mab/9107
p-ERK1/2 (Thr202/Tyr204, #4370P, Cell Signaling) - https://www.cellsignal.com/products/primary-antibodies/phospho-p44-42-mapk-erk1-2-thr202-tyr204-d13-14-4e-xp-rabbit-mab/4370
IκB (#4814, Cell Signaling) - https://www.cellsignal.com/products/primary-antibodies/ikba-l35a5-mouse-mab-amino-terminal-antigen/4814
p-IRF3 (S396, #29047, Cell Signaling) - https://www.cellsignal.com/products/primary-antibodies/phospho-irf-3-ser396-d6o1m-rabbit-mab/29047
IRF3 (#4302, Cell Signaling) - https://www.cellsignal.com/products/primary-antibodies/irf-3-d83b9-rabbit-mab/4302
JNK (#9252S, Cell Signaling) - https://www.cellsignal.com/products/primary-antibodies/sapk-jnk-antibody/9252?site-search-type=Products&N=4294956287&Ntt=9252s%2C&fromPage=plp&_requestid=1990959
p-JNK (Thr183/Tyr185, #4671S, Cell Signaling) - https://www.cellsignal.com/products/primary-antibodies/phospho-sapk-jnk-thr183-tyr185-98f2-rabbit-mab/4671
LC3B (#2775S, Cell Signaling) - https://www.cellsignal.com/products/primary-antibodies/lc3b-antibody/2775
NBR1 (16004-1-AP, Proteintech) - https://www.ptglab.com/products/NBR1-Antibody-16004-1-AP.htm
NDP52 (12229-1-AP, Proteintech) - https://www.ptglab.com/products/CALCOCO2-Antibody-12229-1-AP.htm
p-NF-κB (S536, #3033S, Cell Signaling) - https://www.cellsignal.com/products/primary-antibodies/phospho-nf-kb-p65-ser536-93h1-rabbit-mab/3033?site-search-type=Products&N=4294956287&Ntt=3033s&fromPage=plp&_requestid=1991104
Optineurin (10837-1-AP, Proteintech) - https://www.ptglab.com/products/OPTN-Antibody-10837-1-AP.htm
p38 (#9212S, Cell Signaling) - https://www.cellsignal.com/products/primary-antibodies/p38-mapk-antibody/9212?site-search-type=Products&N=4294956287&Ntt=9212s&fromPage=plp&_requestid=1991144
p-p38 (Thr180/Tyr182, #4511S, Cell Signaling) - https://www.cellsignal.com/products/primary-antibodies/phospho-p38-mapk-thr180-tyr182-d3f9-xp-rabbit-mab/4511?site-search-type=Products&N=4294956287&Ntt=4511s&fromPage=plp&_requestid=1991167
p62 (PM066, MBL) - https://www.mblbio.com/bio/g/dtl/A/index.html?pcd=PM066
Tyrosine hydroxylase (T2928, Sigma) - https://www.sigmaaldrich.com/catalog/product/sigma/t2928?lang=en&region=US
NQO-1 (11451-1-AP, Proteintech) - https://www.ptglab.com/products/NQO1-Antibody-11451-1-AP.htm

Same clones of mouse CD16/CD32 (#14-0161-82, Invitrogen), CD45 Monoclonal Antibody (I3/2.3) conjugated with APC-Cyanine7 (#A15395, Invitrogen), CD11b Monoclonal Antibody (M1/70) conjugated with PerCP-Cyanine5.5 (#45-0112-82, Invitrogen) were used in other papers for microglia/macrophage isolation (CD45 in PMID:25327891, CD11b in PMID:29789522, CD16/CD32 in PMID: 22590615).

# Eukaryotic cell lines

Policy information about cell lines

**Cell line source(s)**

HEK293T cell were purchased from ATCC.

**Authentication**

The cell line used in this study was fully authenticated by the vendor, ATCC (VA, USA).
STR Profile (https://www.atcc.org/Products/All/CRL-3216.aspx#specifications)
- CSF1PO: 11,12
- D13S317: 12,14
- D16S539: 9,13
- D5S818: 8,9
- D7S820: 11
- TH01: 7, 9.3
- TPOX: 11
- vWA: 16,19
- Amelogenin: X

**Mycoplasma contamination**

All cell lines tested negative for mycoplasma contamination.

**Commonly misidentified lines**
(See ICLAC register)

No commonly misidentified cell lines were used.

# Animals and other organisms

Policy information about studies involving animals; ARRIVE guidelines recommended for reporting animal research

| | |
|---|---|
| Laboratory animals | 8-12 weeks-old male mice (C57Bl/6J background) for AAV injection experiments<br>C57BL/6N-Tg(Thy1-SNCA)15Mjff/J(#017682)<br>B6.B10ScN-Tlr4lps-del/JthJ mice (Tlr4-KO, #007227)<br>B6J.B6N(Cg)-Cx3cr1tm1.1(cre)Jung/J mice (Cx3cr1Cre, #025524)<br>B6.129P2(Cg)-Cx3cr1tm2.1(cre/ERT2)Litt/WganJ mice (Cx3cr1CreER-IRES-Eyfp in this study, #021160) were purchased from Jackson Laboratory (Bar Harbor, ME).<br>Atg7flox/flox mice and p62-KO mice were kindly gifted from Dr. Masaaki Komatsu (Tokyo, Japan).<br>Atg14flox/flox mice were Dr. Herbert W.Virgin (Washington University School of Medicine, St. Louis, MO).<br>2-6months-old mice were used for breeding and 1month-old mice were used for genotyping.<br>Mice were maintained in rooms with 12 light/12 dark cycle, 23°C, 40-60% humidity, and water accessible at all times. |
| Wild animals | No wild animals were used in this study. |
| Field-collected samples | No field-collected samples were used in this study. |
| Ethics oversight | All animal procedures were approved by Icahn School of Medicine at Mount Sinai Animal Care and Use Committee (IACUC-2015-0046). |

Note that full information on the approval of the study protocol must also be provided in the manuscript.

# Flow Cytometry

## Plots

Confirm that:

☒ The axis labels state the marker and fluorochrome used (e.g. CD4-FITC).

☒ The axis scales are clearly visible. Include numbers along axes only for bottom left plot of group (a 'group' is an analysis of identical markers).

☒ All plots are contour plots with outliers or pseudocolor plots.

☒ A numerical value for number of cells or percentage (with statistics) is provided.

## Methodology

| | |
|---|---|
| Sample preparation | CD45/CD11b-positive microglia were isolated from adult mice brain using Percoll-gradient method followed by Flow Cytometry. |
| Instrument | BD FACSAria II SORP |
| Software | FACSDiva 8.0.1 (BD) was used for data collection and FCS express 6 flow research software was used for visualization. |
| Cell population abundance | Among microglia-enriched population (gate1 in supplementary figure 2), about 2% cells were shown CD11b(high) and CD45(intermediate)-positive. At the initial experiment, we tested the purity of microglia by comparing EYFP-positive cells from cx3cr1CreER/Eyfp, a microglia-specific cre/Eyfp line, (described in detail on method) and by performing the RT-qPCR using primers against known microglia markers. |
| Gating strategy | Microglia-enriched population by Percoll-mediated fractionation (Gate1, distinct from cell debris by size) were selected, single cell population was gated, and cd11b/cd45-positive cells were collected. Detail gating strategy is provided in supplementary figure 2 and method section. |

☒ Tick this box to confirm that a figure exemplifying the gating strategy is provided in the Supplementary Information.
